# Supplementary material for: Neural evidence for age-related differences in representational quality and strategic retrieval processes
Source: Neurobiol Aging. 2019 Dec;84:50–60. doi: 10.1016/j.neurobiolaging.2019.07.012 (PMC6805220; doi:10.1016/j.neurobiolaging.2019.07.012)
Supplement: Supplementary Materials [file mmc1.docx]

Supplementary Data


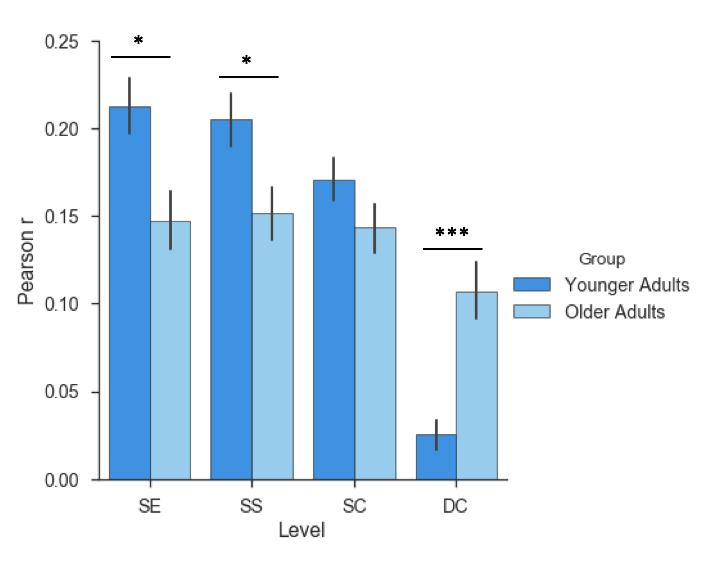


*Figure S1: Pattern similarity at encoding as a function of event relatedness, including subsequently remembered pairs only. SE = Same exemplar; SS = Same Subcategory, SC = Same Category; DC = Different Category. Relative to younger adults, older adults exhibit reduced pattern similarity for related events, coupled with increased pattern similarity for events that are more distinct. Error bars represent standard error of the mean. ***p* < .001; * *p < .05*


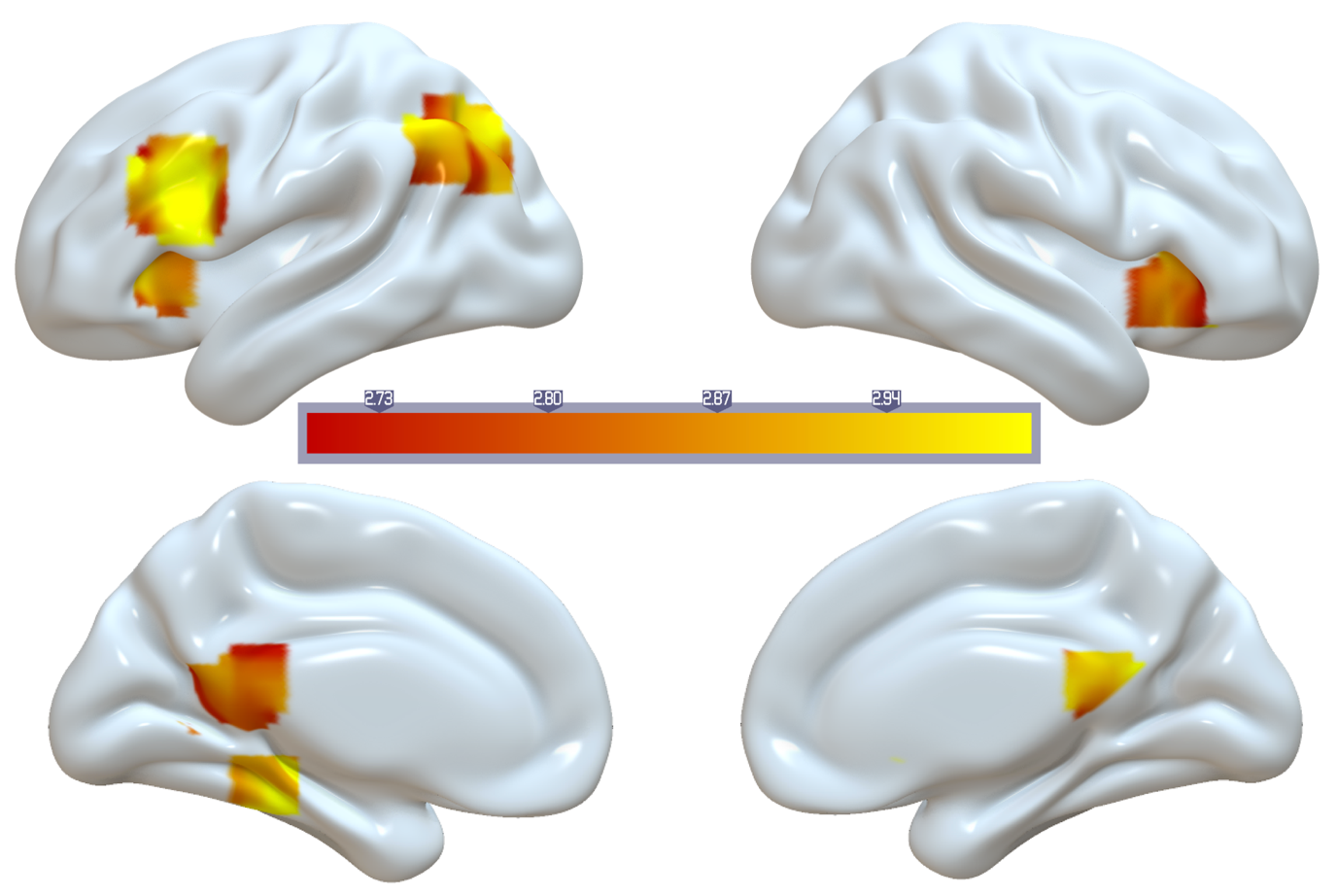


*Figure S2: Univariate Activity: Age x Condition (Hits > CRs) interaction. Whole brain t-maps thresholded at p < .005 uncorrected. Height threshold T = 2.7; Extent threshold k = 5 voxels.*


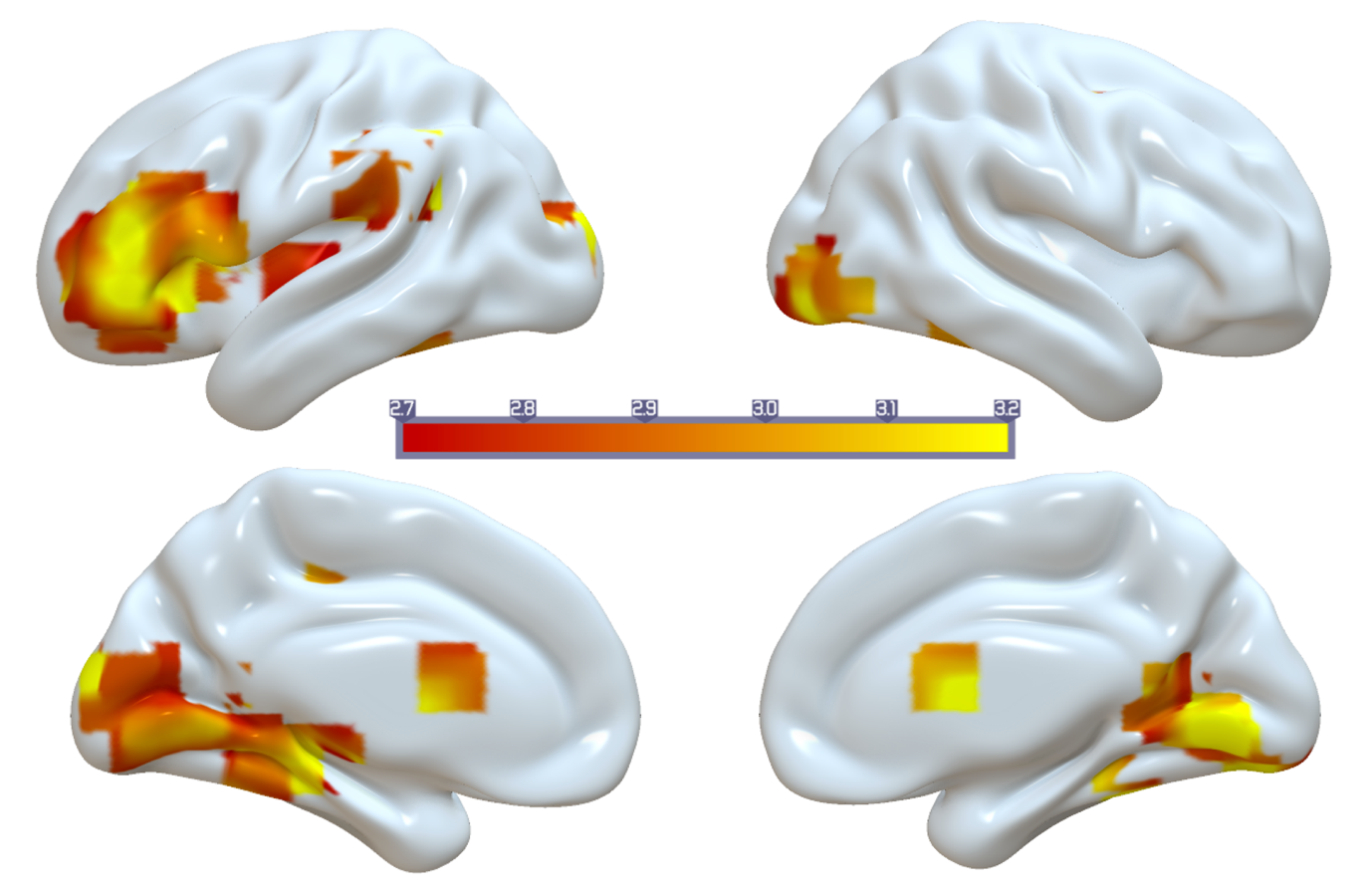


*Figure S3: Hippocampus-whole brain connectivity: Age x Condition (Hits > CRs) interaction. Whole brain t-maps thresholded at p* < *.005, uncorrected. Height threshold T = 2.7; Extent threshold k = 5 voxels.*

*Table S1: Regional BOLD activity during Hits and CRs. Uncorrected whole brain results thresholded at p <* .005. *Asterisk (*) indicates effects that survive FWE p <* .05

| **Region** | **Voxels** | **MNI Coordinates (x, y, z)** | | | **Peak *t* value** |
| --- | --- | --- | --- | --- | --- |
| ***Hits > CRs*** |  |  |  |  |  |
|  |  |  |  |  |  |
| **L hippocampus*** | 468 | -27 | -30 | -12 | 7.96 |
| **L superior medial frontal gyrus*** | 1262 | -6 | 51 | 6 | 7.65 |
| **L lingual gyrus*** | 282 | -12 | -78 | -9 | 7.51 |
| **L inferior parietal cortex*** | 351 | -54 | -45 | 39 | 7.37 |
| **R cuneus*** | 163 | 12 | -93 | 21 | 7.24 |
| **R middle occipital gyrus*** | 71 | 42 | -81 | 24 | 6.82 |
| **R hippocampus*** | 186 | 27 | -18 | -18 | 6.76 |
| **L middle cingulate cortex*** | 393 | 0 | -18 | 39 | 6.33 |
| **R supramarginal gyrus** | 147 | 57 | -30 | 45 | 6.28 |
| **R insula**  **L inferior temporal gyrus**  **R middle temporal gyrus**  **L middle temporal gyrus**  **L insula** | 37  15  15  20  19 | 27  -54  54  57  -36 | 18  -9  -30  -51  -6 | -15  -27  -6  3  18 | 5.87  5.01  4.81  4.64  4.63 |
| ***CRs > Hits*** |  |  |  |  |  |
| **R lingual gyrus***  **L cuneus**  **L superior frontal gyrus**  ***Group X Condition***  **L inferior frontal gyrus (triangularis)**  **L inferior parietal lobe**  **L insula**  **L precuneus** | 160  56  52  106  25  19  19 | 18  -6  -24  -36  -33  -30  -6 | -75  -93  -9  21  -69  27  -48 | -9  9  60  27  39  3  12 | 7.11  5.00  4.68  3.55  3.17  3.11  2.96 |

*Table S2: Hippocampal Connectivity during Hits and Correct Rejections. Uncorrected whole brain results thresholded at p <* .005

| **Region** | **Voxels** | **MNI Coordinates (x, y, z)** | | | **Peak *t* value** |
| --- | --- | --- | --- | --- | --- |
| ***Hits > CRs***  *No suprathreshold clusters*  ***CRs > Hits***  **L pallidum**  **R temporal Pole**  **L temporal Pole**  **L amygdala**  **R caudate**  **R inferior frontal gyrus (orbitalis)**  ***Group X Condition***  **L inferior frontal gyrus (triangularis)**  **L fusiform gyrus**  **L superior occipital gyrus**  **L inferior occipital gyrus** | 48  85  36  15  16  17  306  28  12  24 | -9  54  -48  -15  18  48  -45  -21  -15  30 | 6  6  15  -6  -9  27  -42  -39  -90  -87 | -3  -12  -24  -18  24  -6  12  -12  12  -6 | 4.47  4.16  4.14  3.94  3.66  3.36  3.84  3.58  3.39  3.37 |
